# Mammary Gland Structures Are Not Affected by an Increased Growth Rate of Yearling Ewes Post-Weaning but Are Associated with Growth Rates of Singletons

**DOI:** 10.3390/ani11030884

**Published:** 2021-03-19

**Authors:** Emmanuelle Haslin, Rene A. Corner-Thomas, Paul R. Kenyon, Adrian J. Molenaar, Stephen T. Morris, Hugh T. Blair

**Affiliations:** 1 School of Agriculture and Environment, Massey University, Palmerston North 4474, New Zealand; r.corner@massey.ac.nz (R.A.C.-T.); p.r.kenyon@massey.ac.nz (P.R.K.); s.t.morris@massey.ac.nz (S.T.M.); h.blair@massey.ac.nz (H.T.B.); 2 AgResearch Ltd., Grassland Research Centre, Palmerston North 4474, New Zealand; adrian.molenaar@agresearch.co.nz

**Keywords:** ewe lamb, gland cistern, parenchyma, ultrasonography, ewe hogget

## Abstract

**Simple Summary:**

Increased growth rates of ewe lambs between three and seven months of age can potentially have negative impacts on mammary development and milk production, affecting their capacity to wean a lamb as yearling ewes. This experiment was designed to examine the impacts of an increased growth rate of ewes between weaning and their first breeding at seven months of age on mammary development using ultrasonography and to establish if mammary ultrasound measures could be indicators of growth of yearling ewe progeny. Mammary measures were taken in late pregnancy, early lactation and weaning in 59 single-bearing yearling ewes either preferentially fed and achieving 47.9 kg at breeding at seven months of age, or fed to achieve 44.9 kg at breeding. Mammary measures did not differ between live-weight gain treatments, indicating no evidence of negative effects on mammary development of yearling ewes. Some mammary measures, however, were positively associated with the growth of the progeny to weaning suggesting that ultrasonography has the potential to identify yearling ewes that would wean heavier lambs.

**Abstract:**

The experiment aimed to examine the impacts of an increased growth rate of ewes between three and seven months of age on udder development using ultrasound and to establish whether ultrasonography could be used to identify ewe mammary structures that may be indirect indicators of singleton growth to weaning. Udder dimensions, depths of gland cistern (GC), parenchyma (PAR) and fat pad (FP) were measured in late pregnancy (P107), early lactation (L29), and at weaning (L100) in 59 single-bearing yearling ewes selected from two treatments. The ‘heavy’ group (n = 31) was preferentially fed prior to breeding achieving an average breeding live-weight of 47.9 ± 0.38 kg at seven months of age. The ‘control’ group (n = 28) had an average breeding live-weight of 44.9 ± 0.49 kg. Udder dimensions, GC, PAR and FP did not differ between treatments. Lamb growth to L100 was positively associated (*p* < 0.05) with PAR at P107 and GC at L29. There was no evidence of negative effects of the live-weight gain treatments on udder development of yearling ewes as measured by ultrasonography. The results suggest that this ultrasound method has the potential to identify pregnant yearling ewes which would wean heavier singletons.

## 1. Introduction

A major determinant for achieving puberty and successful breeding of yearling ewes at seven months of age is the attainment of 40–70% of mature live-weight [1,2]. Yearling ewes that weighed 40 to 45 kg at breeding had greater performance than those bred at 35 kg or below, therefore Kenyon et al. [2] recommended a minimum live-weight of yearling ewes of 40 kg at breeding. Further, heavier live-weights at breeding have been shown to improve the reproductive performance of yearling ewes resulting in a greater number of yearling ewes mated during the breeding period, increased fertility rate, litter size, and lambing percentage [2,3]. Using this combined knowledge, farmers aim to feed their Romney-type yearling ewes to achieve suitable growth rates post-weaning to ensure they reach live-weights greater than 40 to 45 kg at breeding at seven months of age. Increased growth rates prior to puberty, however, have been reported to have negative impacts on mammary gland development and milk production in yearling ewes [4,5]. McCann et al. [6] reported that yearling ewes grown at higher rates between weaning and breeding at seven months of age produced less milk than yearling ewes with lower growth rates, indicating an impairment of the mammary gland development and function. Farmers, therefore, need to balance the desire for heavier live-weights at breeding to improve yearling ewe reproductive performance while limiting any potential negative impacts on lactation performance and growth of the progeny to weaning.

Yearling ewes have a period of accelerated growth of parenchymal mammary tissue, called allometric phase, between two and five months of age [7,8]. During this period, the ductal network of the mammary gland expands extensively into the mammary fat pad [9]. The development of the ductal network during this period will determine future alveolar development, and therefore future milk production [5,10]. A high plane of nutrition prior to puberty has been reported to reduce the development of parenchyma [5,8] and increase fat accumulation in the fat pad [8,9], which combined may explain the reported subsequent lower milk production [5,11].

Ewe mammary internal structures can be visualized using ultrasonography [12]. Specifically, ultrasound has been used to investigate the mammary parenchyma [13,14] and the mammary gland cistern (*sinus lactiferous*) [15,16]. In dairy ewes, mammary morphology and milk production were reported as traits of interest in genetic selection, leading to an increase in mammary size and milk yield compared to non-dairy breeds [17,18]. In addition, currently, most studies that have examined mammary structures using ultrasonography have utilised dairy breed ewes [12] with only a small number of studies examining dual-purpose meat and wool breeds [15,19]. Ruberte et al. [19] examined the relationship between mammary ultrasound images and mammary anatomy of mature ewes, whereas Caja et al. [15] focused on measures of cistern size using ultrasound and its correlation with milk yield of mature ewes. Currently, no studies have used ultrasound to examine the mammary gland of non-dairy yearling ewes. Over the last 20 years, ultrasound technology has improved, allowing for more detailed examination of the mammary structures through greater image resolution which allows the assessment of the development of the parenchyma and identification of abnormalities in the parenchyma [12]. The present experiment was the first, to these authors’ knowledge, to utilise ultrasound on non-dairy yearling ewes during their first pregnancy and lactation and to investigate the relationship between ultrasound measurements of yearling ewe udders and the growth of their progeny.

The primary objective of this experiment was to investigate the effects of an increased growth rate between weaning (three months of age) and breeding (seven months of age) on mammary gland dimensions and structures of single-bearing Romney yearling ewes during their first pregnancy and lactation using ultrasonography. It was hypothesised that yearling ewes with an increased growth rate between weaning and breeding (heavy) would have a smaller parenchymal area and a greater fat pad area than yearling ewes with a lower growth rate between weaning and breeding (control). The second objective was to develop an ultrasound technique to identify mammary internal structures that could be used as indirect indicators of the growth to weaning of the progeny of yearling ewes. It was hypothesised that the ultrasound measurements of the young dam’s mammary gland in lactation would be correlated with the early growth rate of their progeny.

## 2. Materials and Methods

The experiment was conducted at Massey University’s Riverside Farm (latitude: 40°50′35″ S, longitude: 175°37′55″ E), 10 km north of Masterton, New Zealand. All animal handling procedures were approved by the Massey University Animal Ethics Committee (MUAEC-17/16).

### 2.1. Experimental Design

#### 2.1.1. Background

**Figure 1:**
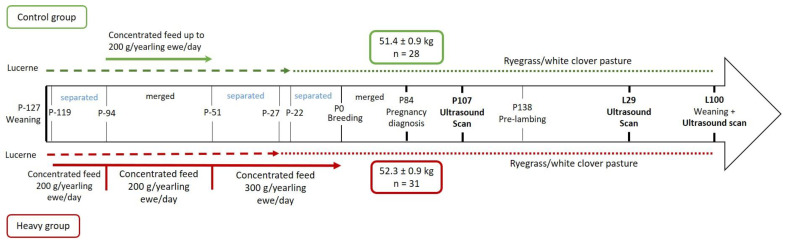
Experimental design from weaning of the yearling ewes (P-127) to weaning of their progeny (L100) (extended of [20]). P: days of pregnancy, L: days of lactation. The lactation period was deemed to have begun after the first lamb of all yearling ewes was born (L1). The green colour (top) corresponds to the control group and the red colour (bottom) corresponds to the heavy group.

As previously described by Haslin et al. [20], at weaning, at approximately 86 days of age (127 days prior to breeding; P-127), 270 twin-born Romney ewe lambs (hereafter called yearling ewes) were allocated to one of the two treatment groups using a stratified random sampling procedure to ensure that the average live-weight of the groups did not differ (28.6 ± 0.16 kg). The intent of this experiment was to bring yearling ewes to different live-weight targets at their first breeding (P0) at seven months of age. The ‘heavy’ group (n = 135) was preferentially fed until breeding (10/05/2018; P0) achieving an average live-weight of 47.9 ± 0.38 kg. The ‘control’ group (n = 135) had an average live-weight of 44.9 ± 0.49 kg at P0. Both groups grazed lucerne sward (*Medicago sativa* L.; heavy for 105 days (P-127 to P-22) and control for 100 days (P-127 to P-27)), then ryegrass (*Lolium perenne* L.) and white clover-based sward (*Trifolium repens* L.) for 22 days heavy (P-22 to P0) and 27 days control (P-27 to P0), and both were offered a cereal-based concentrate feed (CP 10.5%, NDF 17.6%, ADF 7.1%, ME 12.8 MJ/kg DM) pre-breeding (Figure 1). Cereal-based concentrate feed mass offered to the heavy group at a rate of 200 g/yearling ewe/day for 68 days (P-119 to P-51) and 300 g/yearling ewe/day for 51 days (P-51 to P0; Figure 1). Yearling ewes in the control group were offered 200 g/yearling ewe/day for 43 days (P-94 to P-51; Figure 1). Individual animal feed intake was not measured. Pasture allowances were controlled using a rotational grazing system and by differing pre- and post-grazing herbage sward heights. All yearling ewes were managed as a single mob from P0 and bred for two 17-day periods (P0 to P34) to crayon-harnessed Romney rams at a ratio of 1:40 [3]. Yearling ewes were identified as mated in the first 17-day period by recording the presence of a crayon colour mark on their rump [3]. Pregnancy diagnosis was determined at 84 days of pregnancy (P84) using transabdominal ultrasound (Figure 1).

#### 2.1.2. Present Study

Romney yearling ewes from each treatment group were randomly selected at pregnancy diagnosis (approximately 10 months of age) to select only yearling ewes mated during the first 17 days of breeding that were identified as single-bearing (P84; heavy, n = 31, 52.3 ± 0.85 kg and control, n = 28, 51.4 ± 0.85 kg; Figure 1). Only single-bearing yearling ewes were selected as yearling ewes carrying singles are more frequent than those carrying twins [21]. At 138 days of pregnancy (P138), yearling ewes from both treatment groups were randomly assigned to one of four lambing paddocks (average stocking rate 8.02 yearling ewe/ha; heavy n = 8, 6, 9, 8 and control n = 9, 5, 9, 5 per lambing paddock) to ensure yearling ewes from each treatment group in each paddock. Cross-suckling was not controlled, as ewes and lambs are developing an exclusive bond [22] making cross-suckling not frequent. Two yearling ewes in the heavy group died during the lambing period. All yearling ewes lambed within 15 days (1/10/2018 to 16/10/2018). The lactation period was deemed to have begun after the first lamb had been born from all yearling ewes (1/10/2018; L1) and all lambs were weaned at approximately 100 ± 4 days of age (17/01/2019; L100; heavy n = 24 and control n = 24).

From P0 to L100, both groups were managed and grazed together using a rotational grazing system on ryegrass and white clover pasture under commercial New Zealand grazing conditions. The pre-grazing pasture mass during pregnancy and lactation was on average 868 ± 39 and 1265 ± 58 kg DM/ha, respectively. Due to low pasture availability in pregnancy, all yearling ewes were offered lucerne bailage (CP 13.3%, NDF 48.8%, ADF 36.1%, ME 9.6 MJ/kg DM) at a rate of approximately 1.0 kg/yearling ewe/day from P34 to P138.

### 2.2. Animal Measurements

Unfasted live-weights of yearling ewes were recorded at P-127, P0, P84, P107, L29 and L100. Body condition score (BCS) of yearling ewes were recorded at P0, P84, P138, L29 and L100. Lambs were tagged within 18 h of birth, during twice-daily lambing rounds, at which time their date of birth, paddock, sex, dam number and birth weights were recorded. Lambs were reweighed at L29 and L100.

#### 2.2.1. Udder Score and Morphology

Yearling ewe udder scoring, and morphological trait measurements were performed at P107, L29 and L100 by a single trained operator. The scoring system, adapted from Griffiths et al. [23], assessed udder health and included the palpation of both udder halves and teats (Table 1). Yearling ewes were placed in a sitting position to allow access to the udder for palpations.

**Figure 2:**
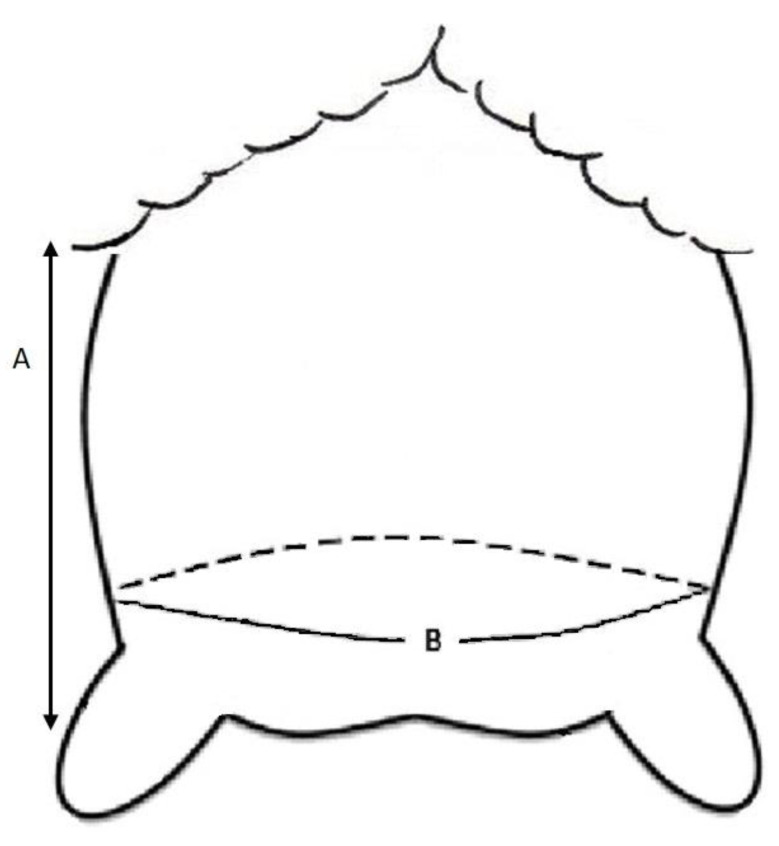
Morphological traits of yearling ewe udder. A. Udder height (cm) measured from outside edge of the udder between the ewe leg and the udder floor; B. Udder circumference (cm) taken above the teats (adapted from [24]).

Morphological traits were measured while yearling ewes were standing and included udder circumference (UC, cm) measured above the teats [24], using a tape (Scrotal Measuring Tape, Shoof international LTD, New Zealand), and the height of each udder half (cm), using a ruler to measure the distance between the rear udder attachment along the outside edge of the udder, and the udder floor [25] (Figure 2). Udder volume (UV, cm^3^) was calculated using UC and an average of udder height (UH) according to Ayadi et al. [25] (1) and (2).

R = UC/2π(1)

UV = π × R^2^ × UH, (2)

where UV = udder volume (cm^3^); π = 3.14159; R = radius (cm); UH = udder height (cm); UC = udder circumference (cm).

#### 2.2.2. Ultrasound Scanning

**Figure 3:**
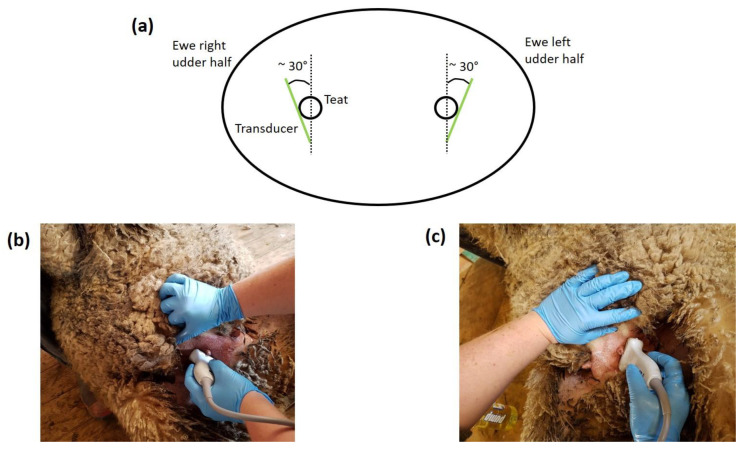
(**a**) Diagram representing the position of the transducer (solid line) applied on the right and left udder halves; (**b**) Picture of an ultrasound scan of the right udder half of a yearling ewe in pregnancy (P107); (**c**) Picture of an ultrasound scan of the left udder half of a yearling ewe in pregnancy (P107).

Ultrasound scans were performed by a single operator, at P107, L29 and L100 (Figure 1). At L29 and L100, ultrasound scans were not conducted for yearling ewes whose lambs had died (heavy n = 5 and control n = 4). At L29 and L100, yearling ewes were separated from their lambs four hours prior to the ultrasound scanning to allow the udder to accumulate milk according to Ruberte et al. [19] and Caja et al. [15]. Yearling ewes were placed in a sitting position (i.e., shearing position; Figure 3b,c) to allow easy access to the udder. Ultrasound scans were performed with an ultrasound scanner fitted with a linear transducer with 5.0–10.0 MHz imaging frequency (Sonosite M-Turbo Ultrasound with L38xi, Bothell, Washington, DC, USA). Vegetable oil was used as a coupling gel. The transducer was applied on the external base of each teat at an approximate angle of 30° from the caudal-cranial axis (Figure 3a) with an inclination of approximately 45° in relation to the teat [26] (Figure 3b,c). A light and consistent pressure was applied to the udder through the transducer to minimise variations related to pressure on the images. There was variability in the position of yearling ewes but, the effects of these variations were minimised by indicating to the handler on which position (on right of left leg) the ewe had to be sited for the ultrasound scan (Figure 3b,c), and by identifying the most representative and consistent mammary structures on the images during the scan prior to capturing the images.

A minimum of three images were taken from each udder half. Images included the gland cistern, mammary parenchyma, putative fat pad and the boundary between the mammary gland and the abdominal wall. One image of suitable resolution per udder half, where all structures were identifiable and present was selected for image processing [27]. Udder halves with an udder palpation score of 4 or 5 (Table 1) at a specific time point (P107, L29 or L100) were considered “abnormal” [23] and were not included in the image selection (heavy: 1 ewe with 1 half and control: 2 ewes with 1 half each).

**Figure 4:**
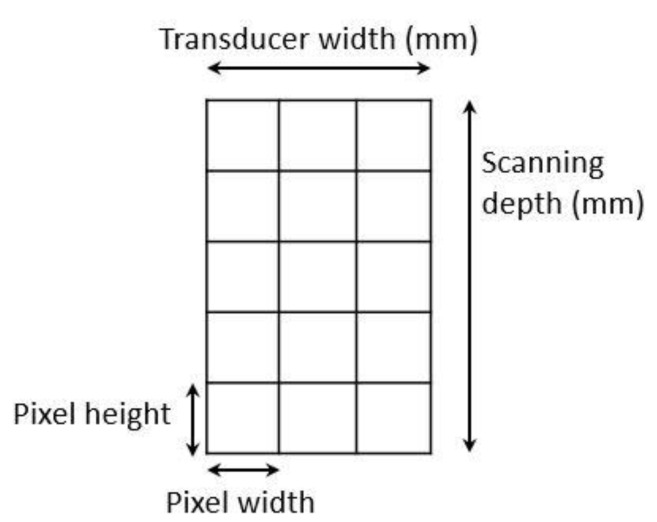
Diagram of an ultrasound image and the dimensions needed to calculate the correspondence between pixels and millimetres.

**Figure 5:**
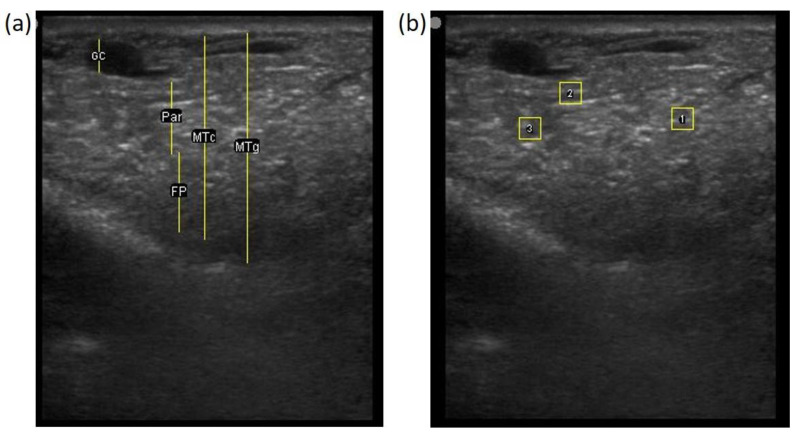
(**a**) Demonstration of delimitations of the mammary total depth conservative (MTc) and generous (MTg), mammary gland cistern (GC), parenchyma (PAR) and the fat pad (FP) and (**b**) Demonstration of randomly positioning of regions of interest (1, 2, 3) in the parenchyma.

The image processing was undertaken using ImageJ software [28] as used by Abràmoff et al. [29]. The scales between pixels and millimetres were calculated based on the number of pixels, the scanning depth (mm), and the transducer width (mm) (Figure 4). This method relies on the ability of the operator to interpret and identify lines on the images. To standardize the assessment compartment depth, drawing templates were created for each time point as used by Molenaar et al. [30] and included four representative images from four different yearling ewes with and without the lines drawn for each compartment (Appendix A). The total depth of mammary gland conservative (MTc) was the smallest likely demarcation (abdominal wall) of the mammary gland (Figure 5a), and total depth of the mammary gland generous (MTg) was the largest likely demarcation of the mammary gland visible on the image [30] (Figure 5a). The MTc, MTg, fat pad (FP), parenchyma (PAR), and gland cistern (GC) depths were estimated at the deepest point for each sub-compartment, excluding the skin layers, using the straight tracer (Figure 5a) and were expressed in millimetres.

To assess the development of the parenchyma at P107, L29, and L100, three regions of interest (ROI; [26]) were randomly drawn in the parenchyma area, each square measured 6.7 mm^2^ (Figure 5b). The brightness of each pixel corresponded to echogenicity and was numerically represented on a scale of 256 levels of grey [31]. Echogenicity is defined as the capacity of tissues to interact and reflect the sound waves of the transducer [32]. This capacity varies with tissues, i.e., liquids have very low echogenicity [32] and fat has greater echogenicity but attenuates as the depth increases [30].

### 2.3. Statistical Analysis

All statistical analyses were conducted using SAS v9.4 (SAS Institute Inc., Cary, NC, USA). Yearling ewes that died (heavy n = 2) or whose lambs died prior to L100 (control n = 4 and heavy n = 5) were excluded from the experiment. The final dataset included 24 yearling ewes and their singletons in each treatment group and a total of 284 images.

Growth of yearling ewes from P-127 to P0 was analysed using a linear mixed model including treatment group (control vs. heavy) as a fixed effect and age at P-127 as a covariate. Live-weight of yearling ewes at P107, L29 and L100 was analysed using a linear mixed model allowing for repeated measures. The model included treatment group, day of measurement (P107, L29 and L100) and their two-way interaction as fixed effects. Lambing date was fitted as a covariate in the model as used by Pettigrew et al. [33]. The BCS of yearling ewes was analysed using a generalized linear model allowing for repeated measures with a Poisson distribution and a log transformation. Treatment group, day of measurement (P0, P84, P138, L29 and L100) and their two-way interaction were included as fixed effects. Growth of the progeny from birth to L29 and from L29 to L100 was analysed using a linear mixed model allowing for repeated measures, and including treatment group, time (birth to L29 and L29 to L100) as fixed effects, date of birth as a covariate and lambing paddock as a random effect.

The ROI grey-scale values, GC, FP, PAR, MTc and MTg of the right and left udder halves were analysed using general linear mixed models allowing for repeated measures. These models included udder half (right vs. left), day of measurement, treatment group and two-way interactions of udder half and day of measurement and treatment group and day of measurement as fixed effects, with a Tukey–Kramer adjustment, lambing date as a covariate and yearling ewe as a random effect. The grey-scale, GC, PAR, MTc, MTg did not differ (*p* > 0.05) between udder halves, therefore, an average of the two halves was calculated for each day of measurement and used in further analyses. For FP, udder halves were significantly (*p* < 0.05) different at L100 and thus the FP measures of the right and left halves at L100, remained separated in the analyses.

To determine the effect of individual yearling ewe live-weight at P0 on the grey-scale values, GC, PAR, FP, MTc and MTg, the linear mixed models were re-run without treatment as a fixed effect and including udder half, day of measurement and their two-way interaction as a fixed effect, lambing date and live-weight at P0 as a covariate, a two-way interaction between live-weight at P0 and day of measurement, and yearling ewe as a random effect.

The residuals were generated using general mixed models. Ewe live-weight, ewe BCS, UV, UH, UC and MTg were adjusted for treatment group and lambing date. In the model, PAR, GC and MTc were adjusted for the treatment group, MTg and lambing date. Lamb growth from birth to L29, L29 to L100 and birth to L100 were adjusted for the treatment group, lambing date, and sex of lamb. Pearson correlations were used to test for linear associations between the residuals of ewe live-weight, UC, UH, UV, GC, PAR, FP, MTc and MTg at each time point (P107, L29, L100) and lamb growth from birth to L29, from L29 to L100 and from birth to L100.

Multiple regression analyses of lamb growth from birth to L29 and from birth to L100 were carried out using general linear models. General linear models were used to examine whether each predictive variable was individually correlated with lamb growth. Predictive variables correlated with lamb growth with *p* ≤ 0.20 were included in the model [34]. Correlations between selected predictive variables were examined to identify high collinearity, resulting in Equations (3) and (4) respectively.

Lamb growth from birth to L29 = GC at L29 + MTc at L29 + BCS at P0(3)

Lamb growth from birth to L100 = PAR at P107 + FP at P107 + Ewe LW at L29 + GC at L29 + MTc at L29(4)

Backward manual variable eliminations were used to select the model that best explained the variation in lamb growth from birth to L29 and to L100 by removing predictive variables with *p* > 0.10. Confounding effects were evaluated after each variable removal and were examined by checking the changes in predictive variable coefficients. Any non-significant predictive variable causing greater than a 20% change in the model coefficients was considered a confounding variable and included in the model [34].

## 3. Results

### 3.1. Growth and Live-Weight

Yearling ewes from the heavy group had greater growth rates between P-127 and P0 than control yearling ewes (*p* < 0.05; 147 ± 4.4 vs. 133 ± 4.4 g/d, respectively) resulting in a tendency for different live-weight at breeding (*p* = 0.09, 47.5 ± 0.71 vs. 45.8 ± 0.71 kg, respectively). Live-weight of yearling ewes, however, did not differ (*p* > 0.05) between treatments at P107, L29 or L100 (Table 2).

Yearling ewe BCS did not differ (*p* > 0.05) between treatment groups at any time point (Table 2). Ewe BCS did not differ (*p* > 0.05) between P0, P84 and P138, which in turn were greater (*p* < 0.05) than BCS at L29 and L100, which did not differ (*p* > 0.05; Table 2).

Lamb live-weights at birth, L29 and L100 (Table 2) and lamb growth from birth to L29, from L29 to L100 (average 340.8 ± 13.5 g/d and 201.5 ± 8.19 g/d, respectively) and lamb growth from birth to L100 [35] did not differ (*p* > 0.05) between treatments.

### 3.2. Udder Scores and Morphology

The udder scores, UH, UC and UV at P107, L29 and L100 were presented in Haslin et al. [35]. Briefly, teat palpation score, udder depth score, the proportion of asymmetric udder and dimensions (UH, UC and UV) did not differ (*p* > 0.05) between treatment groups at any time point. The control group had greater (*p* < 0.01) udder palpation scores at P107 than the heavy group [35].

### 3.3. Ultrasound Measurements

The depth of the gland cistern (GC), parenchyma (PAR), total mammary conservative (MTc), total mammary generous (MTg) and the grey-scale value did not differ between udder halves (*p* > 0.05; data not shown). The depth of the fat pad (FP) did not differ at P107 between udder halves (*p* > 0.05; data not shown), but at L100, the left udder half had a deeper FP than the right udder half (*p* < 0.05; 19.2 ± 0.91 left vs. 15.4 ± 0.99 right).

The depths of GC, PAR, FP, MTc, MTg and the ROI grey-scale values did not differ (*p* > 0.05) between treatment groups at any time point (Table 3). Live-weight at P0, irrespective of treatment group, had no effect (*p* > 0.05) on GC, FP, MTc, MTg and ROI grey-scale values but negatively impacted PAR at P107 (*p* < 0.05; estimate −0.20 mm).

The depth of GC, PAR, MTg and MTc were greater at L29 (*p* < 0.001) than L100 which was, in turn, greater (*p* < 0.001) than P107, irrespective of treatment groups. The depth of FP was greater at L100 than at P107, irrespective of treatment groups (*p* < 0.001; Table 3).

### 3.4. Correlations between Udder Measurements, Ewe Live-Weight, Ewe BCS and Lamb Growth

At P107, UC was positively correlated with UV, UH and PAR (*p* < 0.05), and GC and PAR were negatively associated with FP (*p* < 0.01; Table 4). BCS of yearling ewes at P138 was positively associated (*p* < 0.01) with FP at P107 and live-weight at P107, and negatively associated (*p* < 0.05) with PAR at P107 (Table 4). At L29, UV was positively correlated with UH and UC (*p* < 0.05), and PAR was negatively correlated with GC (*p* < 0.05). Live-weight of yearling ewes at L29, irrespective of treatments, was positively associated with BCS at L29 (*p* < 0.01) and UC (*p* < 0.05; Table 5). At L100, UV was positively correlated with UH, UC, FP of left half (*p* < 0.05), UH was positively associated with UC and FP of the left half (*p* < 0.05). Live-weight of yearling ewes was positively associated (*p* < 0.001) with BCS of yearling ewes at L100 (Table 6) At L100, FP of the left half was negatively correlated with PAR (*p* < 0.01; Table 6).

Lamb growth from birth to L29, irrespective of treatments, was positively associated with GC at L29 (*p* < 0.05; Table 5) and FP at L100 on the right half (*p* < 0.05; Table 6). Lamb growth from birth to L29 to L100 was positively associated with PAR at P107 (*p* < 0.05; Table 4) and FP at L100 on the left half (*p* < 0.05; Table 6) but negatively associated with FP at P107 (*p* < 0.05; Table 4). Lamb growth from birth to L100 was positively associated with PAR at P107 (*p* < 0.01; Table 4), GC at L29 (*p* < 0.05) but negatively associated with PAR at L29 (*p* < 0.05; Table 5).

### 3.5. Multiple Regression of Lamb Growth

The best model explained 12.2% of the variation in lamb growth from birth to L29 included only the effect of GC at L29 (Lamb growth from birth to L29 = 261.4 (±35.1) + 4.9 (±1.9) GC at L29; estimate (±SE)). The difference between a yearling ewe with an average GC at L29 and a GC in the 90th percentile was 6.6 mm (Table 7), resulting in a 32.3 g/d difference in lamb growth from birth to L29.

For the period birth to L100, the best model explained 37.6% of the variation in lamb growth and included the effect of PAR at P107, yearling ewe live-weight (LW) at L29 and GC at L29 (Lamb growth from birth to L100 = 37.4 (±48.5) + 7.2 (±1.9) PAR at P107 + 2.0 (±0.65) LW at L29 + 1.2 (±0.63) GC at L29). The difference between a yearling ewe with an average PAR at P107 and a PAR in the 90th percentile was 2.6 mm (Table 7), resulting in 18.7 g/d in lamb growth from birth to L100. The difference between a yearling ewe with an average GC at L29 and a GC in the 90th percentile was 6.6 mm (Table 7) resulting in 7.9 g/d in lamb growth from birth to L100.

## 4. Discussion

### 4.1. Treatment Effects

The first objective of this experiment was to investigate the effects of increasing growth rates of yearling ewes between weaning and breeding on mammary gland dimensions and internal structures. It was hypothesised that yearling ewes with an increased growth rate would have a reduced mammary parenchymal and a greater fat pad area compared with yearling ewes with a lower growth rate. Despite differences in yearling ewe growth rate between weaning and breeding, depth of the gland cistern, parenchyma, fat pad and total depth of the mammary gland conservative and generous did not differ between treatments, which contrast with previous studies [5,8,11]. The differences in yearling ewe growth rates between treatments from weaning to breeding, however, were small (14 g/d) and the magnitude of the growth (less than 150 g/d on average) was lower than those achieved in previous studies (i.e., from 173 to 305 g/d; [8,11]). The small differences in the growth rate of yearling ewes between treatments in the present experiment (14 g/d) may have impacted the ability of the treatments to alter mammary gland development. There are also differences in breeds between studies that may also explain the difference between the results of the present experiment and previous studies (Hampshire Down [8], Suffolk crossed Dorset and Suffolk [11], Dorset [5]). In the present experiment, there was no evidence of negative impacts on mammary gland development of Romney-type yearling ewes achieving live-weight gains of approximately 147 g/d between their weaning at three months of age and their first breeding at seven months of age and reared under New Zealand conditions.

No differences were found between the right and the left udder half for total depths of the mammary gland, depths of the gland cistern and parenchyma. This finding is consistent with previous studies [13,14]. At weaning, however, the fat pad differed between udder halves. This difference could be explained by a preference of the lamb for one udder half, which may have been over-stimulated [36], in particular for ewes rearing a singleton. Overstimulation of one udder half may lead to an early partial udder involution of the non-preferred udder half resulting in changes in the mammary tissues [37].

### 4.2. Associations between Udder Measurements and Lamb Growth

The second objective of this experiment was to develop an ultrasound technique to identify mammary internal structures that could be used as indirect indicators of lamb growth to weaning. Mammary parenchymal depth in late pregnancy measured by ultrasound was moderately and positively associated with lamb growth to weaning. Singletons born to yearling ewes with a large parenchyma in late pregnancy being 1832 g heavier at weaning at 100 days of age than singletons born to yearling ewes with an average parenchyma in late pregnancy. The mammary parenchyma includes the secretory tissue involved in the production and secretion of milk [38,39] with the number of secretory cells determining milk production [40,41]. Thus, a deeper parenchyma in pregnancy could indicate a greater number of secretory cells and potentially greater milk production. Strzetelski et al. [32] reported in primiparous dairy heifers that the percentage of secretory tissue measured by ultrasound was highly positively correlated with milk production. While the impact of the parenchymal depth in late pregnancy on singleton growth to weaning was moderate, the results suggested that the ultrasound method could potentially be used as a technique to identify pregnant yearling ewes that will wean heavier singletons.

The gland cistern depth in early lactation was moderately and positively correlated to lamb growth from birth to early lactation and to weaning. Singletons born to yearling ewes with a large gland cistern in early lactation being 938 g and 790 g heavier at 29 and 100 days of age respectively, than singletons born to yearling ewes with an average gland cistern in early lactation. The mammary gland cistern is the cavity that milk drains into between milkings or suckling events [38]. Ewes with larger cisterns were reported to produce more milk than ewes with smaller cisterns [15,16,42]. It is likely, therefore, that yearling ewes with larger gland cisterns in early lactation would have greater milk production than those with smaller cisterns. The associations between ultrasound measurements and milk production in non-dairy yearling ewes, however, are still unknown. These results again suggested that ultrasound has the potential to identify yearling ewes that would wean heavier singletons. Further research is warranted to confirm these findings and investigate the use of ultrasound to examine the association between mammary gland, milk production and lamb growth in non-dairy yearling ewes.

The ultrasound scan in late pregnancy was easier to perform, as lactating yearling ewes and lambs had to be separated and were off feed for four hours pre-scanning, whereas late-pregnant yearling ewes did not require a waiting period prior to scanning. The measurement of the parenchyma in late pregnancy relies on the ability of the operator to identify the demarcation between tissues which can be difficult due to the image resolution and the attenuation of signal as the scanning depth increases [30]. The measure of the gland cistern did not so much rely on the operator ability, as the gland cistern appears clearly as black on the image [15,30]. The precise measure of the parenchyma and gland cistern with this ultrasound technique could not be performed at scanning as the correspondence between millimetres and pixels varies depending on the scanning depth. The ultrasound technique used in this experiment would therefore be challenging to apply on larger flocks of yearling ewes. More research is required to improve the ultrasound technique for its potential application on larger flocks.

## 5. Conclusions

This experiment was the first to use ultrasound to investigate the relationship between udder measurements and the growth of progeny of non-dairy yearling ewes during pregnancy and lactation. The depth of the mammary parenchyma in late pregnancy and of the gland cistern in early lactation were indicators of growth from birth to weaning of singletons. Under the conditions of this experiment, there was no evidence of negative effects of the differing live-weight gain treatments between three and seven months of age on mammary gland development of Romney-type yearling ewes during their first pregnancy and lactation as measured by ultrasonography. The results of the association between lamb growth and mammary ultrasound measures suggest that the ultrasound technique used in this experiment has the potential to identify pregnant yearling ewes which would wean heavier singletons. More research is needed to investigate the use of ultrasound to examine the associations between mammary ultrasound measurements, milk production and lamb growth in non-dairy yearling ewes.

## Figures and Tables

**Table 1:** Description of the traits and scores used to assess udder health of yearling ewes in late pregnancy and lactation (adapted from [23]).

Score	Description
Udder palpation ^a^	
1	Diffuse soft consistency
2	Diffuse firm consistency
3	Soft consistency with nodule(s)—lumps or grainy texture
4	Firm consistency with nodule(s)—lumps or grainy texture
5	Diffuse hard consistency
Teat palpation ^a^	
1	Soft consistency
2	Thickened teat orifice
3	Hard consistency
4	Teat orifice obstruction

^a^ Ewes examined in a sitting position.

**Table 2:** Effect of time (P107, P138, L29, L100) and treatment group (control vs. heavy) on yearling ewe live-weight and body condition score (BCS) in pregnancy (P107, P138), early lactation (L29) and at weaning (L100) and lamb live-weight in early lactation (L29) and at weaning (L100). Least square means ± s.e for live-weight and least-square means (95% confidence intervals) for BCS.

Parameters	Pregnancy (P107)	Pregnancy (P138)	Early Lactation (L29)	Weaning (L100)
Ewe live-weight (kg)				
Control (n = 24)	52.3 ± 0.81 ^a^	58.5 ± 0.87 ^b^	61.7 ± 1.21 ^c^	62.0 ± 1.26 ^c^
Heavy (n = 24)	52.4 ± 0.81 ^a^	57.8 ± 0.87 ^b^	60.8 ± 1.21 ^c^	60.2 ± 1.26 ^c^
Ewe BCS				
Control (n = 24)		2.77 (2.64–2.90) ^b^	2.69 (2.53–2.85) ^a^	2.65 (2.48–2.82) ^a^
Heavy (n = 24)		2.69 (2.58–2.80) ^b^	2.46 (2.30–2.63) ^a^	2.42 (2.24–2.60) ^a^
Lamb live-weight (kg)				
Control (n = 24)			15.0 ± 0.52 ^a^	29.9 ± 0.68 ^b^
Heavy (n = 24)			15.1 ± 0.52 ^a^	29.5 ± 0.68 ^b^

^a,b,c^ Means within rows with different superscripts are significantly different (*p* < 0.05).

**Table 3:** Effect of time (P107, L29, L100) and treatments (control vs. heavy) on depths of the mammary gland cistern (GC), parenchyma (PAR), fat pad (FP), the total mammary depth conservative and generous (MTc and MTg), and the grey-scale value of regions of interest (ROI) in the parenchyma (least-square means ± SEM).

Descriptor	Pregnancy (P107)	Early Lactation (L29)	Weaning (L100)	*p* Values (Time)	*p* Values (Treatment)	*p* Values (Treatment × Time)
Control group (n = 24)						
GC (mm)	3.57 ± 0.88 ^a^	16.5 ± 0.88 ^c^	9.62 ± 0.87 ^b^	<0.001	0.082	0.472
PAR (mm)	10.2 ± 0.41 ^a^	46.1 ± 1.32 ^c^	18.9 ± 0.74 ^b^	<0.001	0.600	0.002
FP (mm)	10.3 ± 0.45 ^a^	- ^1^	RS 14.7 ± 1.36 *^,b^ and LS 19.7 ± 1.28 *^,b^	<0.001	0.886	0.970
MTc (mm)	25.6 ± 0.56 ^a^	63.1 ± 1.06 ^c^	44.2 ± 0.98 ^b^	<0.001	0.091	0.063
MTg (mm)	32.2 ± 0.94 ^a^	65.5 ± 0.93 ^c^	49.4 ± 0.96 ^b^	<0.001	0.151	0.519
ROI (grey scale value)	88.0 ± 2.10	93.6 ± 2.79	88.3 ± 2.23	0.390	0.544	0.401
Heavy group (n = 24)						
GC (mm)	4.37 ± 0.88 ^a^	18.2 ± 0.87 ^c^	12.0 ± 0.87 ^b^	<0.001	0.082	0.472
PAR (mm)	8.81 ± 0.41 ^a^	45.8 ± 1.31 ^c^	21.6 ± 0.73 ^b^	<0.001	0.600	0.002
FP (mm)	10.4 ± 0.45 ^a^	- ^1^	RS 16.2 ± 1.43 *^,b^ and LS 18.7 ± 1.31 *^,b^	<0.001	0.886	0.970
MTc (mm)	25.2 ± 0.56 ^a^	63.5 ± 1.06 ^c^	47.7 ± 0.94 ^b^	<0.001	0.091	0.063
MTg (mm)	32.3 ± 0.93 ^a^	67.0 ± 0.93 ^c^	51.4 ± 0.93 ^b^	<0.001	0.151	0.519
ROI (grey scale value)	91.7 ± 2.10	91.5 ± 2.78	90.7 ± 2.22	0.390	0.544	0.401

RS = Right udder half; LS = Left udder half; ^1^ The mammary fat pad was not detected on L29 images; ^a,b,c^ Within row, means with different superscripts are significantly different (*p* < 0.05). * The depth of the fat pad (FP) differed (*p* < 0.05) between the right and left udder half at L100.

**Table 4:** Correlation coefficients of residuals of udder volume (UV), circumference (UC), height (UH), gland cistern (GC), parenchyma (PAR) and fat pad (FP) at P107, live-weight of yearling ewes (Ewe LW) in pregnancy (P107) ^1^, body condition score of yearling ewes (Ewe BCS) in late pregnancy (P138), lamb growth from birth to weaning (Birth to L100), birth to early lactation (Birth to L29) and early lactation to weaning (L29 to L100).

Descriptor	UC	UH	GC	PAR	FP	Ewe LW (P107)	Ewe BCS (P138)	Birth to L100	Birth to L29	L29 to L100
UV	0.738 ***	0.910 ***	0.193	0.243	−0.213	−0.016	−0.038	0.041	−0.163	0.175
UC		0.408 **	0.251	0.315 *	−0.229	−0.062	−0.114	−0.041	−0.056	−0.009
UH			0.109	0.175	−0.168	−0.013	−0.007	0.052	−0.176	0.200
GC				0.040	−0.448 **	−0.026	−0.153	−0.110	−0.156	−0.0009
PAR					−0.625 ***	−0.369 **	−0.314 *	0.373 **	0.091	0.331 *
FP						0.257	0.373 **	−0.374 **	−0.177	−0.246
Ewe LW (P107)							0.413 **	0.005	0.126	−0.115
Ewe BCS (P138)								−0.017	0.019	−0.030
Birth to L100									0.480 ***	0.649 ***
Birth to L29										−0.351 *

* *p* < 0.05; ** *p* < 0.01; *** *p* < 0.001; ^1^ Data for treatment groups were pooled, as no differences (*p* > 0.05) were identified.

**Table 5:** Correlation coefficients of residuals of udder volume (UV), circumference (UC), height (UH), gland cistern (GC) and parenchyma (PAR) at L29, body condition score and live-weight of yearling ewes (Ewe BCS and Ewe LW) in early lactation (L29) ^1^, lamb growth from birth to weaning (Birth to L100), birth to early lactation (Birth to L29) and early lactation to weaning (L29 to L100).

Descriptor	UH	UC	GC	PAR	Ewe LW (L29)	Ewe BCS (L29)	Birth to L100	Birth to L29	L29 to L100
UV	0.648 ***	0.649 ***	0.006	−0.012	0.254	−0.144	−0.100	−0.010	−0.085
UH		−0.100	0.170	−0.145	0.056	−0.097	−0.052	0.007	−0.043
UC			−0.160	0.128	0.289 *	−0.052	−0.003	0.008	−0.009
GC				−0.976 ***	0.011	−0.017	0.298 *	0.284 *	0.071
PAR					0.032	0.029	−0.324 *	−0.272	−0.105
Ewe LW (L29)						0.500 ***	0.180	0.113	0.108
Ewe BCS (L29)							0.171	0.183	0.093

* *p* < 0.05; *** *p* < 0.001; ^1^ Data for treatment groups were pooled, as no differences (*p* > 0.05) were identified.

**Table 6:** Correlation coefficients of residuals of udder volume (UV), circumference (UC), height (UH), gland cistern (GC), parenchyma (PAR) and fat pad from the right (FP Right) and left (FP Left) udder halves at L100, body condition score and live-weight of yearling ewes (Ewe BCS and Ewe LW) at weaning (L100) ^1^, lamb growth from birth to weaning (Birth to L100), birth to early lactation (Birth to L29) and early lactation to weaning (L29 to L100).

Descriptor	UH	UC	GC	PAR	FP Right	FP Left	Ewe LW (L100)	Ewe BCS (L100)	Birth to L100	Birth to L29	L29 to L100
UV	0.771 ***	0.876 ***	0.022	−0.181	0.161	0.317 *	−0.091	−0.112	0.138	−0.088	0.226
UH		0.394 **	0.150	−0.152	0.111	0.309 *	−0.206	0.007	0.006	−0.137	0.135
UC			−0.021	−0.121	0.153	0.200	0.0003	−0.101	0.204	−0.058	0.264
GC				−0.291 *	−0.226	−0.132	0.009	−0.068	0.111	0.194	−0.045
PAR					−0.210	−0.333 *	0.144	−0.062	0.106	−0.105	0.216
FP Right						−0.230	−0.214	−0.067	0.051	0.344 *	−0.233
FP Left							−0.151	−0.026	0.122	−0.280	0.379 *
Ewe LW (L100)								0.664 ***	0.040	0.072	−0.017
Ewe BCS (L100)									−0.202	−0.171	−0.068

* *p* < 0.05; ** *p* < 0.01; *** *p* < 0.001; ^1^ Data for treatment groups were pooled, as no differences (*p* > 0.05) were identified.

**Table 7:** Descriptive statistics of the depths of the gland cistern (GC), parenchyma (PAR) and mammary fat pad (FP) in late-pregnancy (P107), early lactation (L29) and weaning (L100), irrespective of treatment groups.

Descriptor	Minimum	10th Percentile	Mean	90th Percentile	Maximum
GC (mm)					
P107	1.9	2.4	4.0	7.1	8.3
L29	4.2	8.5	17.2	23.8	30.7
L100	4.3	5.4	10.8	16.2	25.8
PAR (mm)					
P107	5.7	6.8	9.5	12.1	17.2
L29	28.8	37.2	46.0	56.6	60.6
L100	12.3	16.6	20.2	26.3	29.9
FP (mm)					
P107	6.0	8.2	10.4	13.2	15.8
L100-Left	2.3	10.1	19.2	27.1	30.9
L100-Right	4.0	7.5	15.4	24.5	28.7

Left: Left udder half; Right: Right udder half.

## Data Availability

The data presented in this experiment are available within the article.

## Appendix A. Drawing Templates of Mammary Ultrasound Images in Late Pregnancy, Early Lactation and Weaning in Yearling Ewes

**Table A1:** Drawing template of mammary ultrasound images in late pregnancy (107 days of pregnancy; P107) in four different yearling ewes.

Time Point and Scanning Depth	Raw Image	Measurement of Udder Structures	Depth (mm)
113 days of pregnancy (P113) Scanning depth 4.7 cm			MT generous: 28.9 mm MT conservative: 25.9 mm Gland cistern: 3.3 mm Parenchyma: 10.9 mm Fat Pad: 10.0 mm
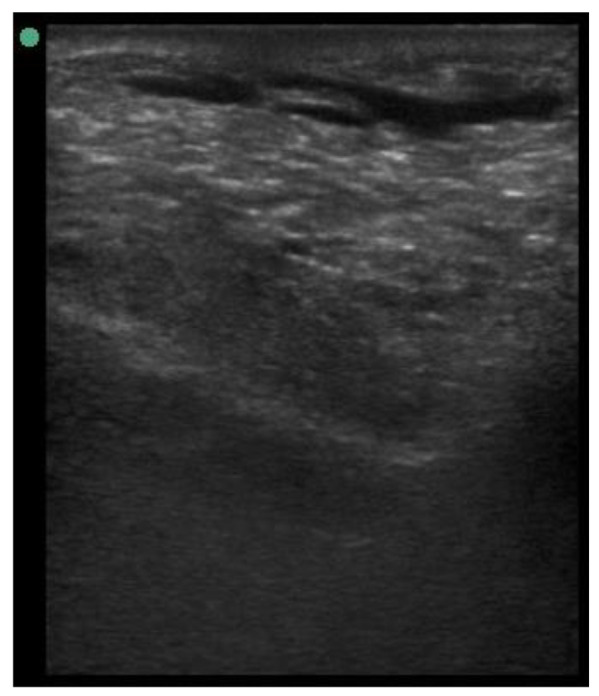
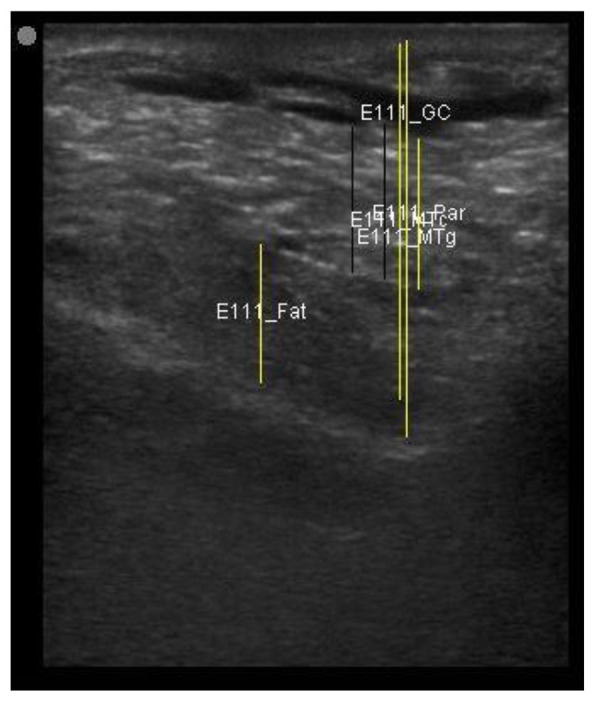
109 days of pregnancy (P109) Scanning depth 4.7 cm			MT generous: 36.8 mm MT conservative: 27.2 mm Gland cistern: 4.7 mm Parenchyma: 15.4 mm Fat Pad: 5.5 mm
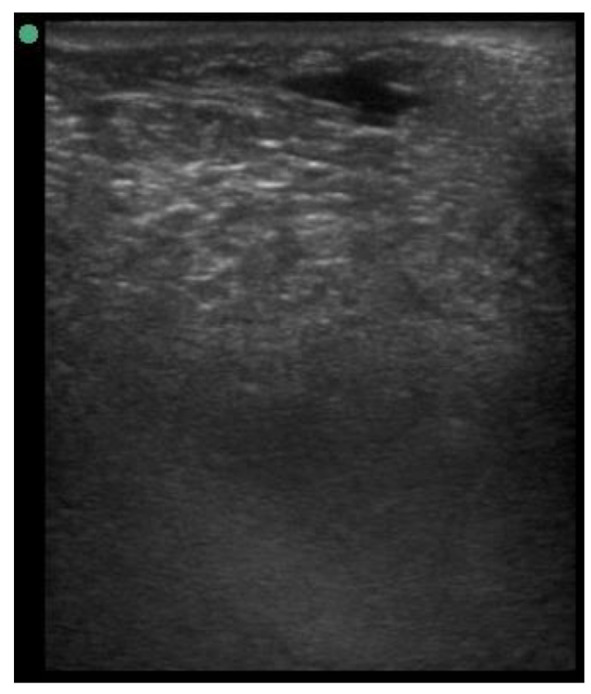
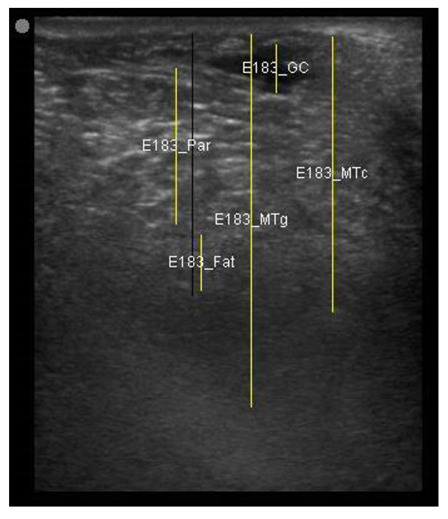
107 days of pregnancy (P107) Scanning depth 4.7 cm			MT generous: 31.7 mm MT conservative: 22.3 mm Gland cistern: 4.5 mm Parenchyma: 8.3 mm Fat Pad: 7.8 mm
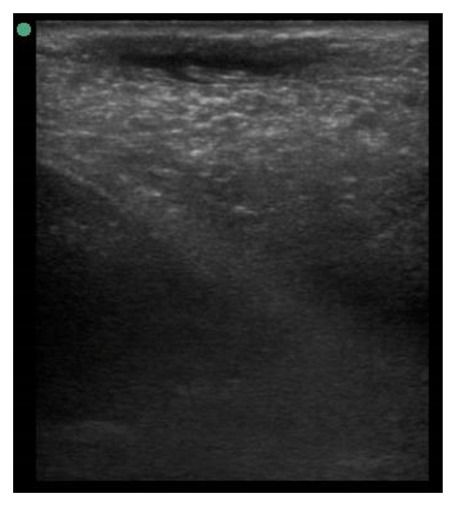
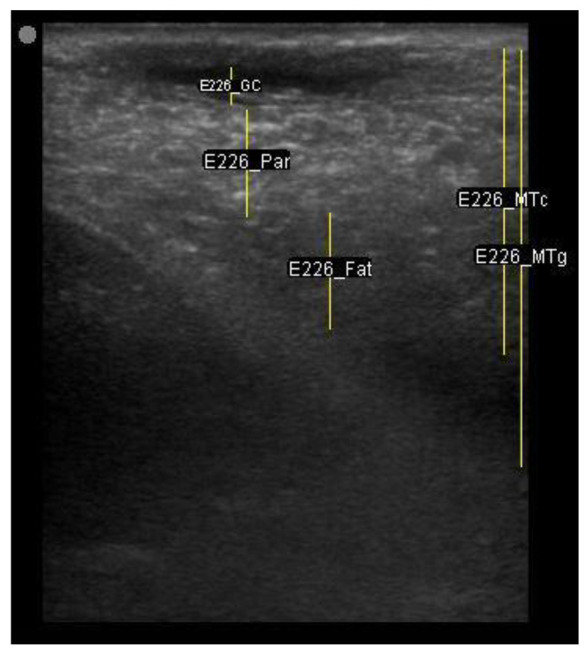
107 days of pregnancy (P107) Scanning depth 4.7 cm			MT generous: 32.2 mm MT conservative: 25.3 mm Gland cistern: 3.6 mm Parenchyma: 12.4 mm Fat Pad: 7.9 mm
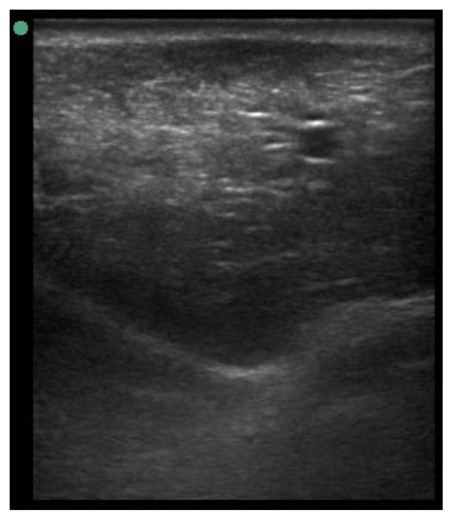
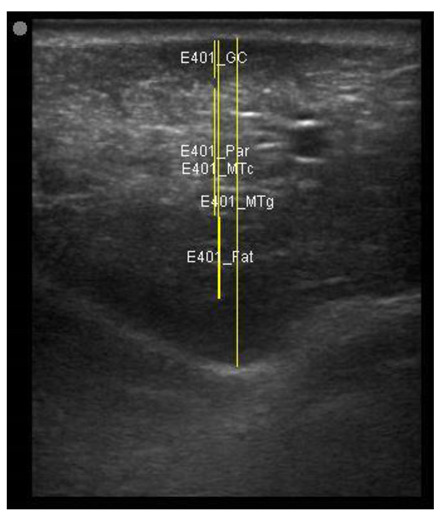

MT: Total depth of mammary gland.

**Table A2:** Drawing template of mammary ultrasound images in early lactation (29 days of lactation; L29) in four different yearling ewes.

Time Point and Scanning Depth	Raw Image	Measurement of Udder Structures	Depth (mm)
32 days of lactation (L32) Scanning depth 7.3 cm			MT generous: 70.6 mm MT conservative: 67.2 mm Gland cistern: 17.6 mm Parenchyma: 49.6 mm
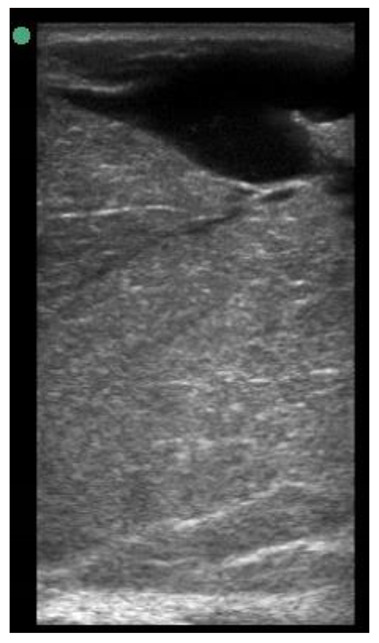
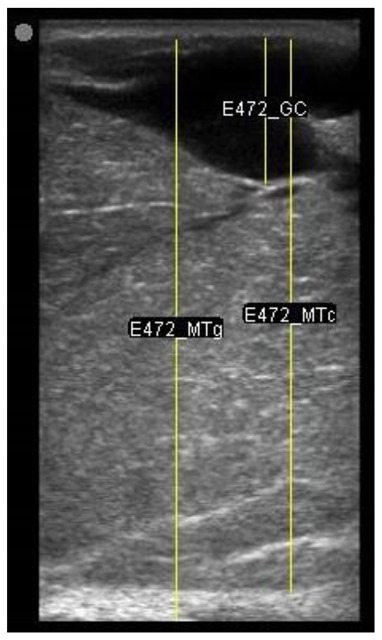
26 days of lactation (L26) Scanning depth 7.3 cm			MT generous: 68.9 mm MT conservative: 63.9 mm Gland cistern: 22.8 mm Parenchyma: 41.1 mm
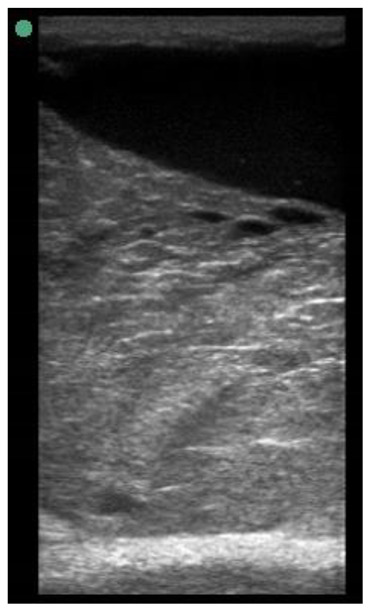
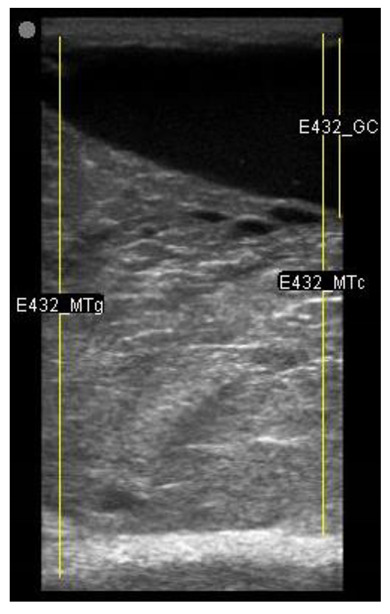
34 days of lactation (L34) Scanning depth 7.3 cm			MT generous: 70.8 mm MT conservative: 67.4 mm Gland cistern: 14.5 mm Parenchyma: 52.9 mm
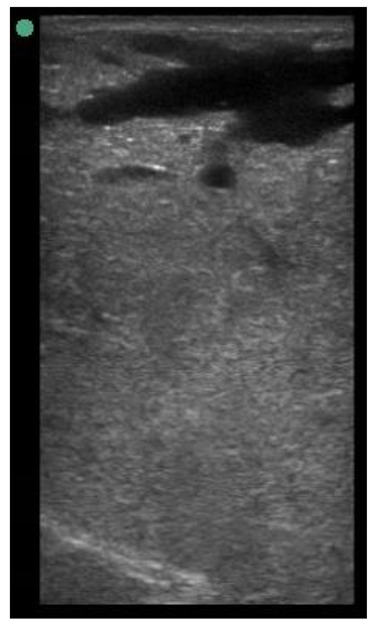
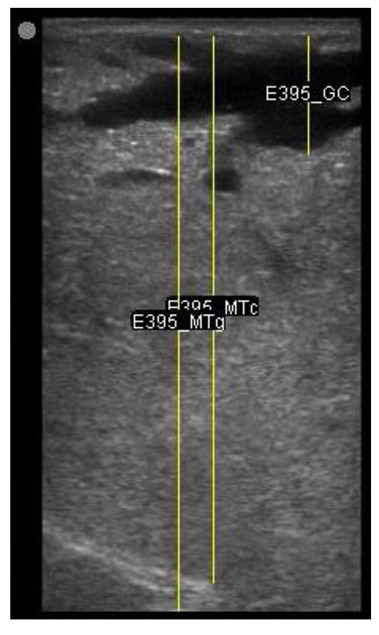
26 days of lactation (L26) Scanning depth 7.3 cm			MT generous: 68.5 mm MT conservative: 65.0 mm Gland cistern: 8.1 mm Parenchyma: 56.9 mm
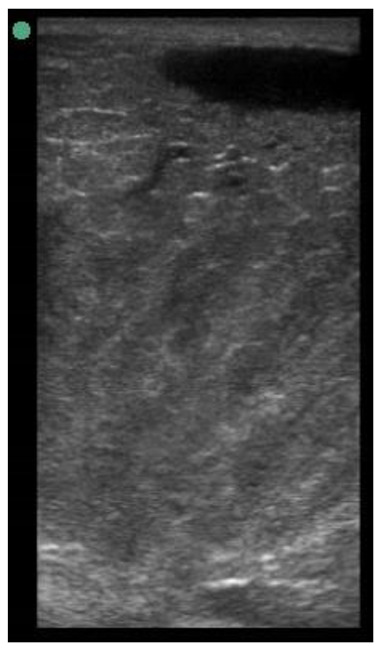
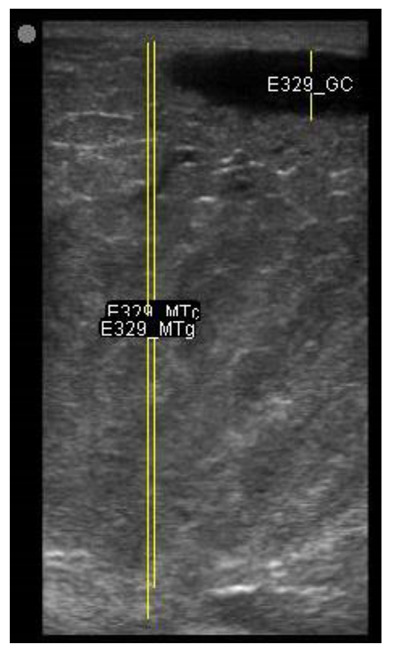

MT: Total depth of mammary gland.

**Table A3:** Drawing template of mammary ultrasound images at weaning (100 days of lactation; L100) in four different yearling ewes.

Time Point and Scanning Depth	Raw Image	Measurement of Udder Structures	Depth (mm)
104 days of lactation (L104) Scanning depth 5.9 cm			MT generous: 53 mm MT conservative: 50.4 mm Gland cistern: 11.9 mm Parenchyma: 15.9 mm Fat Pad: 19.7 mm
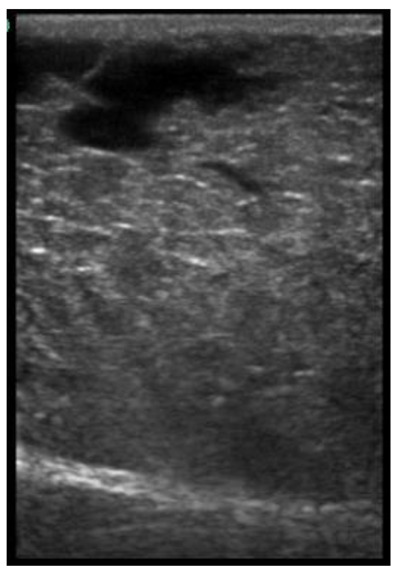
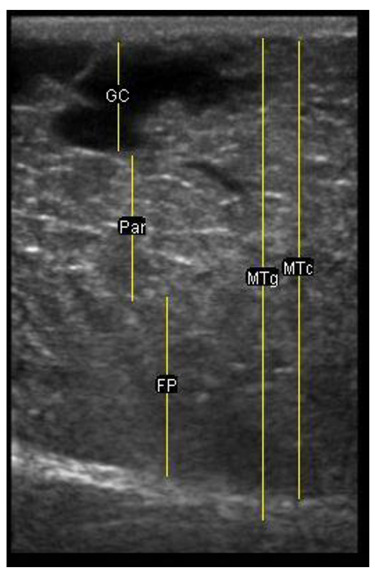
106 days of lactation (L106) Scanning depth 5.9 cm			MT generous: 54.4 mm MT conservative: 50.1 mm Gland cistern: 10.5 mm Parenchyma: 21.1 mm Fat Pad: 15.3 mm
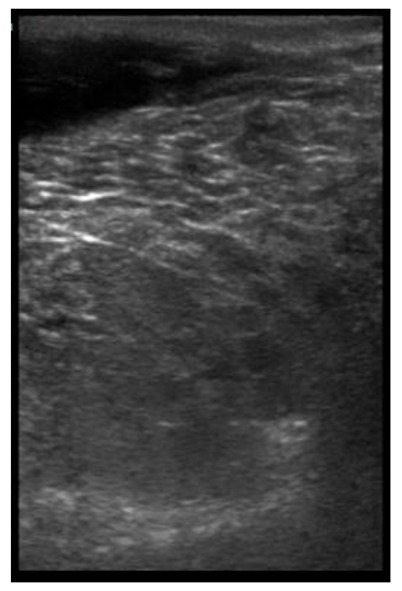
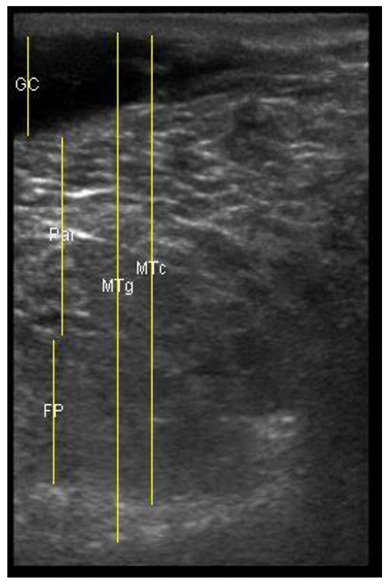
100 days of lactation (L100) Scanning depth 5.9 cm			MT generous: 31.8 mm MT conservative: 28.2 mm Gland cistern: 3 mm Parenchyma: 20.1mm Fat Pad: 8 mm
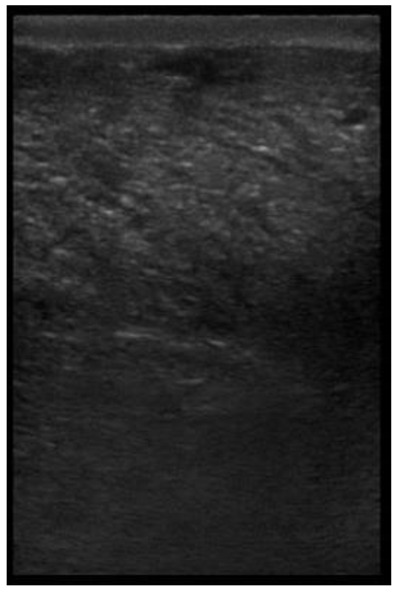
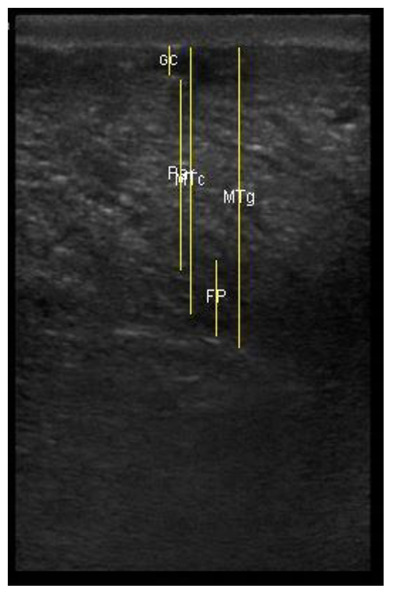
105 days of lactation (L105) Scanning depth 5.9 cm			MT generous: 48.2 mm MT conservative: 39.5 mm Gland cistern: 4.4 mm Parenchyma: 25.1 mm Fat conservative: 11.9 mm Fat generous: 19 mm
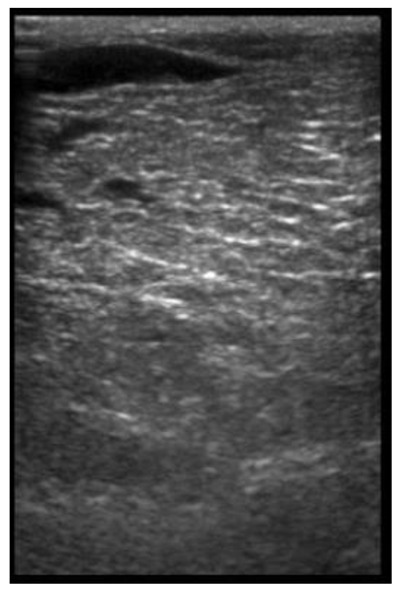
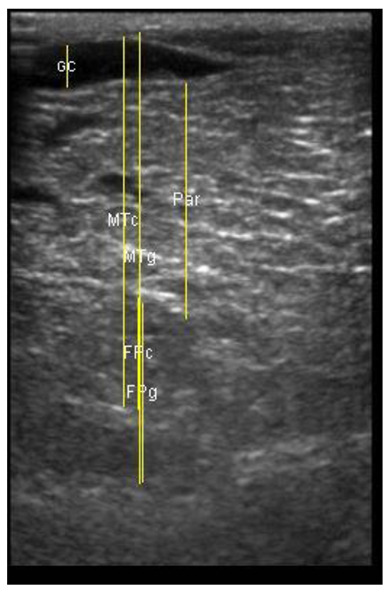

MT: Total depth of mammary glan.
